# Does improving indoor air quality lessen symptoms associated with chemical intolerance?

**DOI:** 10.1017/S1463423621000864

**Published:** 2022-01-12

**Authors:** Roger B. Perales, Raymond F. Palmer, Rudy Rincon, Jacqueline N. Viramontes, Tatjana Walker, Carlos R. Jaén, Claudia S. Miller

**Affiliations:** Department of Family and Community Medicine, Hoffman TILT Program, University of Texas Health Science Center, San Antonio, USA

**Keywords:** chemical intolerance, environmental house calls, indoor air quality, volatile organic compounds

## Abstract

**Aim:**

To determine whether environmental house calls that improved indoor air quality (IAQ) is effective in reducing symptoms of chemical intolerance (CI).

**Background:**

Prevalence of CI is increasing worldwide. Those affected typically report symptoms such as headaches, fatigue, ‘brain fog’, and gastrointestinal problems – common primary care complaints. Substantial evidence suggests that improving IAQ may be helpful in reducing symptoms associated with CI.

**Methods:**

Primary care clinic patients were invited to participate in a series of structured environmental house calls (EHCs). To qualify, participants were assessed for CI with the Quick Environmental Exposure and Sensitivity Inventory. Those with CI volunteered to allow the EHC team to visit their homes to collect air samples for volatile organic compounds (VOCs). Initial and post-intervention IAQ sampling was analyzed by an independent lab to determine VOC levels (ng/L). The team discussed indoor air exposures, their health effects, and provided guidance for reducing exposures.

**Findings:**

Homes where recommendations were followed showed the greatest improvements in IAQ. The improvements were based upon decreased airborne VOCs associated with reduced use of cleaning chemicals, personal care products, and fragrances, and reduction in the index patients’ symptoms. Symptom improvement generally was not reported among those whose homes showed no VOC improvement.

**Conclusion:**

Improvements in both IAQ and patients’ symptoms occur when families implement an action plan developed and shared with them by a trained EHC team. Indoor air problems simply are not part of most doctors’ differential diagnoses, despite relatively high prevalence rates of CI in primary care clinics. Our three-question screening questionnaire – the BREESI – can help physicians identify which patients should complete the QEESI. After identifying patients with CI, the practitioner can help by counseling them regarding their home exposures to VOCs. The future of clinical medicine could include environmental house calls as standard of practice for susceptible patients.

## Introduction

### Chemical intolerance

Increased prevalence of chemical intolerance (CI) has recently been reported by Hojo *et al*. (2018) and Steinemann (2019a). Katerndahl *et al.* (2012) report that twenty percent of primary care patients report adverse reactions associated with low-level exposures to chemical inhalants, foods, and drugs – everyday exposures that do not bother most people and are not generally recognized as toxic. These responses do not appear to be IgE-mediated (Ashford & Miller, 1998). Those affected typically report symptoms such as headaches, mood changes, fatigue, ‘brain fog’, and gastrointestinal problems – common complaints in family medicine practices (Finley *et al*., 2018; Katerndahl *et al*., 2012). But are health care providers aware of how exposures may contribute to these symptoms? Primary care physicians are often the first to see these chemically intolerant individuals whose symptoms defy diagnosis and frustrate patients and practitioners alike. Common symptom triggers for those with CI include indoor air contaminants such as combustion products from gas stoves and smoking, volatile and semi-volatile organic compounds (VOCs and SVOCs) from products like disinfectants, pesticides, air fresheners, fragrances, and chemicals outgassing from new furnishings, paint, carpeting, flooring, glues, and construction materials (Miller & Mitzel, 1997; Miller & Prihoda, 1999a; 1999b; Fanger, 2006; Norbäck, 2020).

### Health and the indoor air environment

The effect of indoor air on individual and population health has been studied for over 40 years (Samet *et al*.,^1997^; Norbäck, 2020). However, many patients and their caregivers are surprised to learn that indoor air is more polluted than outdoor air (Chen & Zhao, 2011). The groundbreaking 1985 Environmental Protection Agency (EPA) ‘TEAM’ study revealed that levels of common organic pollutants were two to five times higher inside homes than outdoors, even in industrialized areas (Wallace *et al.*, 1985). In the 1970s, energy conservation concerns led to construction of more tightly sealed homes and office buildings and a major reduction in fresh air intake (Ashford & Miller, 1998). Consequently, the levels and complexity of indoor air exposures increased.

Currently, there are no requirements, only recommendations, regarding adequate fresh air intake for homes, but several agencies, including the World Health Organization (WHO 2020), the Environmental Protection Agency (EPA, 2021), Centers for Disease Control and Prevention National Institute for Occupational Safety and Health (CDC/NIOSH, 2021), American Society of Heating and Air-Conditioning Engineers (ASHRAE, 2018; 2020), the U.S. Consumer Product Safety Commission (CPSC, 2021) and the U.S. Department of Housing and Urban Development (USD HUD, 2021), have issued practical guidelines or sponsored research on home indoor air quality. Because Americans spend approximately 90% of their day indoors at home, work, or school, indoor air pollutants, *even at low levels*, may affect susceptible individuals first (Jenkins *et al*., 1992; Klepeis *et al*., 2001).

The air inside an individual’s home is of particular importance because air inhaled in the home comprises more than half the body’s lifetime intake (Sundell, 2004). A substantial body of epidemiological evidence links indoor air pollution with health conditions including asthma, COPD, and other respiratory ailments, cardiovascular morbidity, stroke, and cancer (Harada *et al*., 2010; Kelly & Fussel, 2015). The WHO attributes approximately 4.3 million premature deaths to household air pollution (WHO, 2014).

### Indoor volatile organic compounds (VOCs)

Indoor VOCs are chemical compounds present as gases/vapors that are released at indoor temperatures from sources such as carpeting, adhesives, furnishings, vinyl shower curtains, cleaning chemicals, and consumer products such as fragrances, nail polish, cosmetics, new construction and remodeling materials, and air fresheners (Loftness *et al*., 2007; EPA, 2017; Vardoulakis *et al*., 2020).

### VOCs and susceptible individuals

Some groups are especially susceptible to adverse effects stemming from exposures to indoor air pollutants. Those who spend most of their time at home and indoors, such as infants and toddlers, the elderly, and the chronically ill or disabled, are particularly vulnerable (US Consumer Product Safety Commission, 2020). CI is on the rise both nationally and internationally, and people with CI now comprise 20% of the U.S. population (Hojo *et al.*, 2018; Steinemann, 2019a). However, to date, research and public policy regarding indoor air quality (IAQ) have not addressed their needs.

For individuals with CIs, the selection of safe materials or products for the home remains difficult. Materials labeled as environmentally safe may not be compatible with good IAQ (Wasley, 2000). Nussbaumer (2004; 2006) identifies several sources of VOCs that affect those with CI and calls for architects and interior designers to work together on research with the medical community and sensitive individuals.

Agents added to consumer products as fragrance are one recognizable and potentially harmful group of VOCs. In a U.S.-based study, 35% of respondents attributed adverse health effects to fragranced consumer products such as cleaning supplies, air fresheners, fabric softeners, and personal care products (Potera, 2011; Steinemann, 2019b). Among those who report CI, over 80% report difficulty with breathing, headaches, asthma attacks, or mucosal or skin problems with exposure to fragrance. Fragranced consumer products are typically composed of tens to hundreds of compounds, many derived from petrochemicals (Steinemann, 2019b). Prior research shows that as total VOC levels increase, the probability of adverse effects increases including burning and irritation in the eyes, nose, and throat; headaches; nausea; and nervous system effects (ECA-IAQ, 1997; Salthammer 2011; WHO, 2020).

### Home interventions

There is a substantial body of research on home interventions to improve children’s health, particularly asthma (Wu & Takaro, 2007; Colbeck *et al.*, 2010; Bruce & Pope, 2015). Kao *et al.* (2009) describe the historical and future use of medical house calls and their effectiveness for certain key medical issues including wound care, respiratory care and oxygen therapy, bladder and bowel management, enteric and intravenous feeding or therapy.

The Seattle-King County Healthy Homes Project (Kreiger *et al.*, 2005; 2009) was one of the first systematic attempts to reduce indoor air asthma triggers. Two hundred seventy-four low-income households containing a child aged 4–12 years who had asthma were provided in-home environmental assessments, education, support for behavior change, and resources. Over 1-year, they found that a high-intensity(seven visits) versus low-intensity home intervention (one visit) was more effective in improving quality-of-life measures and reducing utilization of asthma-related urgent health services.

A recent systematic review of efforts to improve home air quality and evaluate their effectiveness showed mixed results (Quansah *et al.*, 2017). The more effective interventions have focused on childhood asthma (Croker *et al*., 2011). Numerous investigations have addressed asthma and allergy triggers in patients’ homes and demonstrated that education and home visits can be effective at reducing symptom triggers among susceptible individuals, primarily children (Croker *et al.*, 2011; Le Cann *et al.*, 2017).

Although VOCs have long been recognized as important components of IAQ (Ayoko & Wang, 2018) and much is known about the sources of IAQ, there has been little research on direct home interventions for those who are sensitive to very low-level VOCs (Miller & Ashford, 2000). Recently, Norback *et al.* (2020) emphasize the need for more research on sensitive subgroups (such as those with CIs) and the importance of investigating multiple interactions between various VOCs, mold, and other indoor factors with respect to health effects.

### Our pilot study and hypothesis

We investigate the effectiveness of environmental house calls (EHCs) in reducing the symptoms of individuals with CI. Our context is *the patient-centered medical home*. This model for primary care is an accepted philosophy of health care delivery that encourages providers to achieve excellence and ensure that care is delivered in the right place, at the right time, and in the manner that best suits a patient’s needs (Crabtree *et al*., 2010; Jaen *et al.*, 2010; Stange *et al.*, 2010; Jackson *et al.*, 2013; Miller *et al*., 2014). This philosophy has already been in place in the form of medical house calls primarily serving the elderly and others with chronic physical illnesses or unstable health (Hayashi & Leff, 2012; Ruben *et al*., 2015).

However, little has been done specifically for those with CIs. Our pilot study was conducted as an initial attempt to close this gap. We developed the Environmental House Call (EHC) with three primary goals in mind: (1) assisting individuals who have CI with identification of potential home exposures, how to avoid them, and how to reduce or eliminate potential symptom triggers; (2) evaluating compliance through objective pre-/post-VOCs measurements provided by an independent lab; and (3) evaluating any symptom changes associated with changes in VOCs.

We hypothesized that those who adhered to the EHC recommendations would demonstrate a reduction in VOCs and concomitant reduction of CI symptoms.

## Methods

### Recruitment measures

This study was approved by the University of Texas Health Science Center IRB protocol #HSC20150821H. Participants for the Environmental House Call Study were volunteers recruited from the waiting room of the Family and Community Medicine primary care clinic. Qualifying participants were at least 18 years old. They were first screened for CI using the 3-item Brief Environmental Exposure and Sensitivity Inventory (BREESI), which asks about adverse reactions to chemical inhalants, foods/food additives, and drugs/medications. The validity of the BREESI for effectively screening CI has been demonstrated in two prior studies (Palmer *et al*., 2020, 2021). Individuals who answered ‘yes’ to one or more of these items were invited to complete the 50-question Quick Environmental Exposure and Sensitivity Inventory (QEESI) (Miller, 2001) to confirm CI and assess symptoms.

### Ascertaining chemical intolerance and related symptoms

The QEESI is a validated, self-administrable questionnaire that helps differentiate chemically intolerant individuals from the general population (Miller and Prihoda, 1999a; 1999b). Researchers in more than sixteen countries have used the QEESI, and it has emerged as the reference standard for assessing CI (eg Hojo *et al.*, 2003; Jeon *et al.*, 2012; Skovbjerg *et al*., 2012; Palmer *et al.,* 2021). The QEESI has four scales, but only the Symptom Severity and Chemical Intolerance scales are used to gauge CI. Within each scale, individual items are scored from 0 to 10. The Chemical Intolerance scale lists 10 potential exposures that may be problematic (eg engine exhaust, tobacco smoke, insecticides, gasoline, paint, cleaning products, perfumes, tar, nail polish, new furnishing/construction) and rates for symptom severity (0 = ‘not a problem’ to 10 = ‘severe or disabling problem’).

Similarly, the Symptom Scale rates 10 symptoms on a 10-point Likert type (0 = not at all a problem, 5 = moderate symptoms, 10 = disabling symptoms). The 10 symptoms evaluated on this scale are as follows:

1. Musculoskeletal Symptoms (MS): Problems with your muscles or joints, such as pain, aching, cramping, stiffness, or weakness?
2. Airway or Mucous Membrane Symptoms (AIR/MM): Problems with burning or irritation of your eyes, or problems with your airway or breathing, such as feeling short of breath, coughing, or having a lot of mucus, postnasal drainage, or respiratory infections?
3. Heart/Chest-related Symptoms (COR): Problems with your heart or chest, such as a fast or irregular heart rate, skipped beats, your heart pounding, or chest discomfort?
4. Gastrointestinal Symptoms (GI): Problems with your stomach or digestive tract, such as abdominal pain or cramping, abdominal swelling or bloating, nausea, diarrhea, or constipation?
5. Cognitive Symptoms (COG): Problems with your ability to think, such as difficulty concentrating or remembering things, feeling spacey, or having trouble making decisions?
6. Affective Symptoms (AFF): Problems with your mood, such as feeling tense or nervous, irritable, depressed, having spells of crying or rage, or loss of motivation to do things that used to interest you?
7. Neuromuscular Symptoms (NM): Problems with balance or coordination, with numbness or tingling in your extremities, or with focusing your eyes?
8. Head-related Symptoms (HEAD): Problems with your head, such as headaches or a feeling of pressure or fullness in your face or head?
9. Skin-related Symptoms (SKIN): Problems with your skin, such as a rash, hives, or dry skin?
10. Genitourinary Symptoms (GU): Problems with your urinary tract or genitals, such as pelvic pain or frequent or urgent urination? (For women: or discomfort or problems with your menstrual period?)

Scores greater than or equal to 40 on both scales are *very suggestive* of CI. Scores from 20–39 on one or both scales are *suggestive* of CI. Scores less than 20 on both scales are *not suggestive* of CI (Miller & Prihoda, 1999a; 1999b). Inclusion criteria for participation in the EHCs were QEESI scores >=40 on both the Chemical Intolerance and Symptom Severity scales. In order to evaluate symptom improvements, participants completed the Symptom Scale at baseline and at the 8–12-month follow-up visit.

### Intervention and reference sample

In order to participate in the house calls, participants needed to have a QEESI score indicative of CI (eg *very suggestive*), be willing to allow our intervention team to enter their homes for 5 visits over a 1-year period, and be open to making recommended changes to their home environment.

We screened 745 individual outpatients with the BREESI and 424 completed the QEESI. Forty-three met the EHC study qualifications. Of the 43 who received the first house call, 6 of them were lost to follow-up, with 37 completing the EHC study. Our reference group was comprised of 22 individuals who self-identified as chemically intolerant and who met our QEESI inclusion criteria. They were recruited from online CI patient groups (not from our family practice clinic) and were located throughout the United States. They did not receive the EHC or any other intervention. They had previously completed the QEESI, and we re-administered it approximately 10 months later, contemporaneously with the house calls. Comparing our intervention group scores to this online reference group would demonstrate whether, without any intervention, self-reported symptoms would remain stable over this time.

### EHC intervention

A team led by a Certified Indoor Environmental Consultant (CIEC) performed all home assessments. A total of five visits per home were conducted over a 6- to 10-month period.

*Visit 1*: (1 h) Participants completed a consent form and a pre-questionnaire which included demographics and medical and exposure history. Our team performed a detailed walkthrough assessment. A home evaluation checklist, developed for this study, was used to document the ages, sizes, and physical condition of homes (carpet, construction, furniture, and major outgassing sources), as well as household products used for cleaning and the presence of pets and pests. We photographed pre-intervention conditions including personal care products, cleaning and laundry products, and other potential sources of exposure in the home. No coaching was provided.

*Visit 2*: (2 h) During this visit, initial IAQ sampling was performed. Participants were instructed to keep windows and doors closed for at least 24 h prior to the visit. Active sampling was used to measure specific VOCs using a pump with a charcoal filter in a glass tube for 2-h sampling. Samples were shipped to and analyzed by PRISM analytics (PRISM Analytical, 2020), who use proprietary algorithms to estimate VOC levels (ng/L) and attribute them to various source categories.

During an in-home teaching session, the team discussed indoor air exposures and their health effects with participants and their families and provided preliminary guidance for reducing exposures. Participants received a free starter kit with recommended cleaning supplies and ‘recipes’ for safer, fragrance-free cleaning practices.

*Visit 3:* (1 h) Approximately 1 month after Visit 2, a personalized action plan was presented to participants in their homes. The plan focused on exposures of concern identified by the team during their walkthrough and any sampling results outside normal lab ranges. Our team discussed the plan’s specific guidance for improving indoor air quality and answered any questions. Participants were to implement their action plan over the next 6–10 months.

*Visit 4:* (2 h) The QEESI was re-administered to assess any changes in symptoms. The post-EHC questionnaire assessed participants’ perceived adherence to the EHC interventions in the action plan. All environmental sampling and analyses from Visit 2 were repeated.

*Visit 5:* **(**1 h) A final report including pre-/post-environmental findings was shared with participants.

### Statistical methods

Statistical analysis consisted of comparison of baseline/follow-up change in the total symptom scores between those receiving the EHC and the reference group, and evaluation of symptom improvement in the EHC group as a function of change in VOC levels. The five VOC categories we found most salient in this pilot study were as follows: (1) total volatile organic compounds (TVOC); (2) light solvents; (3) odorants & fragrances; (4) personal care products; and (5) a composite terpene variable representing the averaged levels of limonene, linalool, α-pinene, and β-pinene.

Analysis 1: A paired t-test was used to evaluate symptom improvement from baseline to follow-up comparing the EHC intervention group to the reference group that received no EHC.

Analysis 2. To determine symptom improvements in the intervention group, change scores were created for the Total Symptom Severity score, and for each of the ten symptom subscales. This was calculated by subtracting baseline scores from follow-up scores. Therefore, higher values represent greater symptom reduction. These change scores were grouped into tertile improvement groups: *Low/No improvement* (1st tertile), *Some improvement* (2nd tertile), and *Greatest improvement* (3rd tertile) in symptoms.

To test the hypothesis that symptom improvements were associated with reductions in VOCs, a paired t-test was used to determine the extent of change in VOC levels stratified by the tertile improvement groups. All analyses were performed using SAS software (SAS 2014).

### Statistical power

Sample sizes of 20–30 with a minimum of 12 for pilot studies have been recommended by various research statisticians (Birkett & Day, 1994; Browne, 1995). In this pilot study (*n* = 37 for the intervention group and *n* = 22 for reference group), the power to detect small (.10) or medium effect sizes (.30) in pre-post-measures of VOCs is low (power = .65, using two-tail, alpha = .05, matched pairs t-test). There is 80% power to detect a large effect size (0.5). However, pilot studies are performed prior to definitive trials to provide enough evidence of overall potential intervention benefits (Leon *et al.*, 2011; Lee *et al*., 2014). Schoenfeld (1980) suggests that preliminary hypothesis testing for efficacy could be conducted with a high type I error rate (a false positive rate up to *P* < 0.25). In this study, we accept an alpha in the range of *P* < 0.20, to avoid missing a true effect (Type II error).

## Results

Table 1 shows that the demographics of those receiving the intervention and the reference group were not statistically different. The average age of participants was approximately 55 years old with a preponderance of females (∼80%).

**Table 1.** Sample demographics

	Total	Environmental house call group (*n* = 37)	Reference group with no EHC (*n* = 22)
Demographic			
Age	55.3 (12.4)	54.12 (16.30)^a^	55.8 (10.3)^a^
Percent female	85%	86%^a^	83%^a^
Percent currently married	44%	62.5%^a^	37.5%^a^

Note: Superscripts with the same letter in the two groups signify no statistical difference. e.g., ^a^ EHC = Environmental house call.

### Analysis 1: comparing baseline and follow-up symptom scores between EHC and referent groups

**Figure 1.**
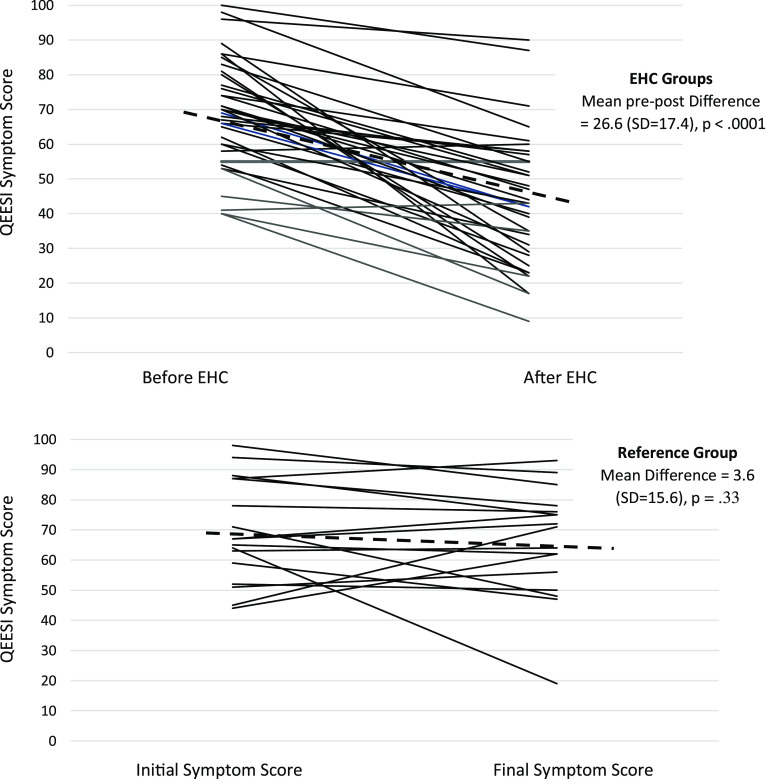
QEESI symptom scores for EHC group before and after house calls and for Reference group over similar timeframe (6–10 months).

Table 2 shows the pre-post-intervention Symptom Severity Scale scores comparing those in the EHC and reference groups. Those receiving the EHC had a significant 26.6-point improvement from baseline on the total QEESI Symptom Severity Scale score (*P* < .0001). The online reference group showed no significant change in Symptom Severity Scale scores (3.6-point improvement from baseline, *P* = .33). Figure 1 shows baseline and follow-up Symptom Severity Scale changes for all 37 EHC participants and the 22 in the reference group. Note the variation among participants. On average, there is a significant change in symptoms in the EHC group, but not all participants improved – some stayed the same, and some got worse. Similarly, in the reference group, there is individual variation, but no average significant change.

**Table 2.** Initial and final total symptom scores comparing those receiving or not receiving an EHC

	Environmental house calls (*n* = 37)			Reference group with no EHC (*n* = 22)		
	Mean	SD	Range	Mean	SD	Range
Baseline symptom severity scores (range 0–100)	69.7	(15.9)	40–100	70.1	(16.6)	44–98
Follow-up symptom scores	43.1	(19.7)	9–90	66.4	(17.8)	19–93
	Mean Difference = 26.6 (SD = 17.4) *P* < .0001			Mean Difference = 3.4 (SD = 15.6) *P* = .39		

EHC = Environmental house call.

During the EHCs, our team watched for exposures and practices that might explain the multi-system symptoms, frequently neurological, that participants reported. Had they noticed tobacco smoking or mold in multiple homes that would have been a red flag. Notably, our team observed near-universal use of potent cleaning chemicals, personal care products, and commercial fragrance products like ‘air fresheners’, incense, scented candles, or ‘plug-ins’ – as many as *ten* of these in a single home.

### Analysis 2: determining whether symptom improvements were associated with significant reductions in VOCs

Table 3 shows pre-post-reductions for (1) TVOCs; (2) odorants and fragrances; (3) light solvents; (4) personal care products; and (5) average terpenes, each stratified into the three improvement groups on the *Total Symptom Scale*: (1) no improvement; (2) some improvement; and (3) most improvement.

**Table 3.** Changes in VOC (ng/L) by QEESI total symptom scale improvement groups

Variable	Total sample		Low/no total symptom improvement		Some total symptom improvement		Greatest total symptom improvement	
	Mean	SD	Mean	SD	Mean	SD	Mean	SD
Total VOC PRE	2113.89	(1507.9)	1991.7	(1821.3)	2250.8	(1489.9)	2168.5	(1259.0)
Total VOC POST	1844.44	(1261.3)	1637.5	(1512.5)	2041.7	(978.1)	1926.9	(1285.8)
Light solvents PRE	54.17^+^	(76.6)	71.8	(123.1)	37.3	(22.0)	51.0^+^	(47.6)
Light solvents POST	35.72	(25.1)	42.8	(31.7)	37.1	(25.1)	28.1	(15.2)
Odorants & fragrances PRE	283.56	(767.3)	474.8	(1306.3)	150.8	(131.3)	222.3	(282.5)
Odorants & fragrances POST	151.25	(245.9)	143.6	(303.4)	158.4	(158.8)	150.9	(262.2)
Personal care products PRE	775.28^**^	(454.7)	586.7	(276.1)	983.3	(632.0)	760.8	(296.9)
Personal care products POST	640.00	(309.0)	556.7	(257.5)	742.5	(3321.0)	688.5	(398.0)
Average terpenes PRE	17.2^**^	(24.89)	9.8	(15.1)	17.7	(17.1)	24.8^+^	(35.1)
Average terpenes POST	12.7	(15.45)	12.0	(19.7)	13.0	(7.7)	14.3	(17.4)

^+^ *P* > .15 & < .20; *P < .15 & > .10; **P < .10 & > .05; ***P < .05.

VOC = volatile organic compound; QEESI = Quick Environmental Exposure and Sensitivity Inventory.

Relative to the other groups, the group demonstrating the greatest total symptom improvement revealed greater change on all five VOC assessments. However, statistically significant changes were observed only for light solvents and terpenes.

Table 4 shows pre-post-VOC reductions for each of the ten symptom subscales stratified by the symptom improvement groups. With a few exceptions, VOC reductions reached or trended toward significance primarily for the group reporting the greatest symptom improvement. Notable among those whose symptoms improved most were reduced levels of light solvents, personal care products, and terpenes.

**Table 4.** Changes in VOC (ng/L) by QEESI subscale improvement groups

Variable	Low/no cognitive symptom improvement		Some cognitive symptom improvement		Greatest cognitive symptom improvement		Low/no head symptom improvement		Some head symptom improvement		Greatest head symptom improvement	
	Mean	SD	Mean	SD	Mean	SD	Mean	SD	Mean	SD	SD	Mean
Total VOC PRE	2103.8	(1622.4)	2074.6	(1588.9)	2178.0*	(1399.9)	2050.9	(1816.3)	2146.7	(1374.4)	2136.9**	(1464.9)
Total VOC POST	2126.1	(1530.9)	1670.0	(1201.0)	1705.0	(978.8)	2119.0	(1704.0)	2083.3	(1221.7)	1391.5	(709.9)
Light solvents PRE	39.3	(39.1)	63.6	(115.4)	61.1***	(49.8)	42.4	(44.4)	37.7**	(28.5)	79.2*	(116.8)
Light solvents POST	52.00	(30.4)	29.0	(15.81)	23.3	(16.2)	57.0	(31.1)	24.2	(12.1)	28.3	(17.3)
Odorants & fragrances PRE	506.3	(1239.1)	152.8	(258.3)	163.8	(202.6)	570.7	(1349.9)	115.1	(136.3)	196.1^+^	(263.5)
Odorants & fragrances POST	208.3	(305.4)	83.1	(82.2)	165.6	(300.0)	210.9	(333.7)	157.3	(271.5)	95.2	(96.2)
Personal care products PRE	703.1	(318.5)	920.7*	(624.9)	680	(316.0)	611.8	(288.4)	756.7	(385.5)	930.8**	(588.7)
Personal care products POST	666.9	(362.6)	655.3	(221.5)	585	(354.8)	691.8	(351.5)	630.8	(328.3)	604.6	(270.0)
Average terpenes PRE	14.8	(13.9)	11.6	(17.3)	27.1**	(38.7)	23.1	(30.4)	17.2	(28.7)	13.1*	(16.9)
Average terpenes POST	16.2	(18.8)	9.1	(6.6)	12.9	(19.5)	16.2	(20.7)	17.1	(16.3)	6.0	(4.5)

Variable	Low/No GI improvement		Some GI improvement		Greatest GI improvement		Low/no affect improvement		Some affect improvement		Greatest affect improvement	
	Mean	SD	Mean	SD	Mean	SD	Mean	SD	Mean	SD	SD	Mean
Total VOC PRE	2119.2	(1935.7)	2246.7	(1525.1)	1962.7*	(926.0)	2438.3*	(2150.8)	1530.0	(625.7)	2373.3	(1287.5)
Total VOC POST	2133.1	(1617.1)	1868.3	(1147.3)	1477.3	(851.3)	1746.7	(1543.7)	1760.0	(1031.8)	2026.7	(1248.8)
Light solvents PRE	37.1	(39.6)	67.3	(120.1)	60.0**	(46.6)	75.3	(121.6)	36.8	(40.5)	50.4**	(37.6)
Light solvents POST	48.2	(31.1)	26	(16.2)	31.6	(20.7)	48.8	(28.3)	27.1	(25.3)	31.3	(16.7)
Odorants & fragrances PRE	449.3	(1249.5)	102.4	(147.1)	285.3***	(297.2)	505.8	(1298.1)	143.8	(157.2)	201.0	(284.6)
Odorants & fragrances POST	275.6	(372.0)	100.3	(101.3)	59.8	(42.8)	176.8	(302.9)	125.0	(156.6	152.0	(273.2)
Personal care products PRE	783.1	(343.0)	895.8*	(678.9)	634.6	(190.1)	771.7	(645.9)	671.7	(286.6)	882.5***	(366.4)
Personal care products POST	704.6	(323.3)	606.7	(283.1)	600.0	(335.0)	576.7	(269.9)	715.0	(364.4)	628.3	(296.1)
Average terpenes PRE	20.0	(29.7)	12.9	(18.6)	18.9*	(27.1)	17.3	(22.6)	9.9	(8.2)	24.4^+^	(35.4)
Average terpenes POST	19.8	(23.0)	10.4	(6.8)	6.6	(4.5)	12.4	(19.5)	11.6	(8.9)	14.2	(17.4)

Variable	Low/No NM improvement		Some NM improvement		Greatest NM improvement		Low/no skin improvement		Some skin improvement		Greatest skin improvement	
	Mean	SD	Mean	SD	Mean	SD	Mean	SD	Mean	SD	SD	Mean
Total VOC PRE	2503.6	(2152.4)	2005.0	(1275.1)	1831.1	(886.7)	2031.7	(1014.8)	2102.9	(1999.2)	2228.0	(1336.9)
Total VOC POST	1880.0	(1540.4)	1900.6	(1154.3)	1701.1	(1210.2)	1620.0	(703.8)	2095.0	(1759.6)	1763.0	(967.8)
Light solvents PRE	39.5	(42.3)	45.0	(41.2)	88.4	(135.9)	76.9	(123. 5)	29.4	(20.0)	61.6*	(43.54)
Light Solvents POST	43.6	(28.2)	31.8	(20.1)	33.0	(29.7)	32.8	(22.1)	37.1	(31.6)	37.3	(20.03)
Odorants & fragrances PRE	595.1	(1340.45)	153.3	(239.3)	134.3	(173.5)	187.7	(264.3)	407.7	(1210.5)	224.8	(218.3)
Odorants & fragrances POST	194.5	(316.6)	137.1	(235.9)	123.7	(176.0)	84.8	(72.7)	189.7	(303.1)	177.2	(294.9)
Personal care products PRE	835.5	(669.8)	736.9	(356.4)	770.0	(312.6)	809.2	(380.5)	728.6	(610.5)	800.0*	(288.5)
Personal care products POST	597.3	(275.6)	654.4	(302.0)	666.7	(384.9)	676. 7	(212.5)	595.0	(371.3)	659.0	(334.4)
Average terpenes PRE	8.9^+^	(6.9)	14.5	(15.0)	24.38*	(34.5)	15.3***	(13.1)	11.4*	(17.7)	28.1^+^	(40.6)
Average terpenes POST	11.7	(20.6)	12.3	(8.3)	13.8	(15.7)	7.1	(5.0)	16.7	(18.8)	14.2	(18.2)

Variable	Low/no musculoskeletal improvement		Some musculoskeletal improvement		Greatest musculoskeletal improvement		Low/no coronary improvement		Some coronary improvement		Greatest coronary improvement	
	Mean	SD	Mean	SD	Mean	SD	Mean	SD	Mean	SD	SD	Mean
Total VOC PRE	2036.4^+^	(1907.5)	1721.0	(722.1)	2432.7	(1589.5)	2707.7^+^	(1908.5)	1582.0	(715.9)	1929.2*	(1396.6)
Total VOC POST	1597.3	(1639.6)	1833.0	(1001.0)	2033.3	(1151.7)	2057.7	(1591.3)	1958.0	(1258.1)	1543.9	(873.4)
Light solvents PRE	76.9	(123.37)	53.8	(57.15)	37.7*	(31.5)	80.8	(121.5)	36.4	(22.7)	41.2	(27.3)
Light solvents POST	38.9	(25.65)	48	(32.7)	25.2	(13.5)	39.4	(31.2)	35.0	(27.9)	32.6	(16.0)
Odorants & fragrances PRE	496.2	(1362.9)	174.5	(170.6)	200.3	(273.6)	540.8	(1246.8)	111.0	(145.2)	159.1	(183.4)
Odorants & fragrances POST	148.5	(317.6)	130.6	(163.0)	167.1	(247.7)	209.4	(311.6)	88.7	(50.3)	141.2	(265.1)
Personal care products PRE	608.2	(328.6)	698.0	(303.5)	949.3**	(567.4)	920.8	(548.0)	658.0	(343.7)	720.0**	(420.1)
Personal care products POST	572.7	(286.6)	671.0	(352.8)	668.7	(308.1)	744.7	(305.3)	661.0	(346.2)	519.2	(259.7)
Average terpenes PRE	8.9^+^	(6.9)	14.5	(15)	25.8**	(33.8)	21.3*	(21)	7.1*	(4.1)	22.4^+^	(34.7)
Average terpenes POST	11.7	(20.7)	12.3	(8.4)	14.7	(15.5)	14.3	(19.3)	10.8	(6.9)	13.5	(16.5)

Variable	Low/no airway improvement		Some airway improvement		Greatest airway improvement		Low/no GU improvement		Some GU improvement		Greatest GU improvement	
	Mean	SD	Mean	SD	Mean	SD	Mean	SD	Mean	SD	SD	Mean
Total VOC PRE	1746.2	(1504.2)	1746.2	(1504.2)	1976.2	(983.8)	1939.3	(1005.3)	1857.8*	(829.7)	2560.8	(2180.8)
Total VOC POST	1491.5	(660.1)	1491.5	(660.1)	1656.9	(1055.4)	1766.0	(964.1)	1265.6	(507.9)	2409.2	(1689.7)
Light solvents PRE	51.0	(50.8)	51	(50.8)	73.23^+^	(115.1)	65.1	(112)	24.9	(12.6)	59.3^+^	(40.48)
Light solvents POST	35.3	(29.2)	35.31	(29.2)	31.38	(25.1)	33.3	(28.4)	33.4	(27.0)	40.2	(18.64)
Odorants & fragrances PRE	162.8**	(168.7)	162.8	(168.7)	102.15	(148.9)	87.2	(77.3)	262.7^+^	(313.8)	517.4	(1240.0)
Odorants & fragrances POST	86.9	(86.1)	86.9	(86.1)	106	(150.6)	97.7	(139.3)	77.9	(67.9)	263.0	(359.8)
Personal care products PRE	651.5	(612.2)	651.5	(612.2)	848.46**	(329.1)	764.0	(322.6)	577.8^+^	(273.8)	928.5	(614.1)
Personal care products POST	541.5	(279.2)	541.5	(279.2)	663.85	(329.6)	741.3	(329.9)	451.1	(259.4)	720.0	(349.1)
Average terpenes PRE	15.7	(21.1)	20.7	(32.2)	18.4	(24.9)	14.5	(15.6)	9.5	(8.5)	28.3*	(37.4)
Average terpenes POST	7.6	(5.3)	24.7	(25.2)	10.7	(9.6)	10.1	(9.0)	8.1	(6.7)	19.5	(22.3)

* *P* < .15 & > .10.

** *P* < .10 & > .05.

*** *P* < .05.

+ *P* > .15 & < .20.

VOC = volatile organic compound; QEESI = Quick Environmental Exposure and Sensitivity Inventory; GI = gastrointestinal symptoms; NM = neuromuscular symptom; GU = genitourinary symptom.

## Discussion

Our goal was to determine whether an EHC aimed at improving indoor air quality corresponded with significant symptom changes for symptomatic CI individuals. The EHC included inspection, measurement, education, and detailed action plans to identify and reduce exposures in the homes of individuals with CI that might be causing their symptoms. Not all participants experienced significant symptom improvements. Improvements were greatest among those who complied and succeeded in reducing their home VOC levels. The VOCs that improved are primarily from consumer products such as personal care and cleaning products. These exposures present easily actionable and accessible targets for clinicians seeking low-cost, low-risk interventions. Patients who may benefit most from such interventions are those suffering from the symptoms listed on the QEESI subscales as cognitive, head-related, gastrointestinal, affective, and musculoskeletal.

Primary care clinicians view individuals holistically, in the contexts of their social and physical environments (Valentijn *et al*., 2013). They are uniquely prepared to recognize and intervene when home exposures may contribute to illness, for example, lead paint in older homes. Poor indoor air quality is an invisible contributor to illness. Interventions for asthma and allergies have received the most recognition. Despite mounting evidence of adverse effects on health, the importance of indoor VOCs, especially for susceptible populations, remains understudied and underappreciated (Klepeis *et al*., 2001; Zhang & Srinivasan, 2020)

Although individualized house calls or air quality monitoring are not yet accessible in standard medical practice, there are simple tools available at low to no cost that can be employed to help patients. The QEESI is a practical clinical tool for assessing symptoms, chemical and other intolerances, and their life impact. Patients can be counseled to avoid salient exposures and track any health changes using the Symptom Star, a graphing tool included with the QEESI. Resources to aid clinicians are available online (www.chemicalintolerance.org). Indoor air consultants who work with chemically intolerant patients must understand the heightened susceptibility these patients have to exposures tolerated by most people and should be able to identify all potential contributing sources and practices.

Although further research is needed to support the clinical value of assessing intolerances and intervening in the home, general advice to reduce home exposures to VOCs should be considered basic preventive practice, given in the spirit of the precautionary principle. These interventions benefit not only the patients, but their entire household, which may include other vulnerable family members such as infants and young children, pregnant women, and those with chronic health conditions. The homes inspected in this study contained a wide range of products that release VOCs (See Table 5).

**Table 5.** Common findings in the homes of participants that may be symptom triggers

Fragranced personal care products	Household products	Scented household products
Soaps	Floor and surface cleaners	Laundry products
Shampoos	Paints/thinners	Detergents
Deodorants	Fragrance-emitting devices (air fresheners and plug-ins)	Fabric softener
Cosmetics	Scented candles/incense	Dryer sheets
Oral hygiene products	Insect repellents	
Hair spray and other hair products	Pesticides	
Lotions/oils	Fragranced garbage/trash bags	
Perfumes/cologne		
Nail polish/remover		

Given that EHCs currently are not widely available, physicians can counsel their chemically intolerant patients, based on our experience, ‘The best odor in a home is no odor. If you smell something, you are inhaling molecules. Find the source and try to remove it’. Remediating an entire household can be challenging. Some chemically intolerant patients have benefitted from designating one or more rooms as a ‘clean air oasis’ where exposures and sources are minimized. There are many resources available to guide this process, including our two-page ‘7 Steps to Creating a Clean Air Oasis’ with safer cleaning ‘recipes’ (available in Spanish and English; see Supplemental Material S1. For other recommended resources see supplement S1).

Although fragrances are not the sole source of VOCs, based upon our research and that of many others, chemically intolerant individuals generally report fragrances as potent symptom triggers (Miller & Mitzel 1997; Ashford & Miller, 1998; Miller & Prihoda, 1999a; 1999b; Potera, 2011; Steinemann, 2019a; 2019b). Indeed, at the outset of our study, 65% of our sample reported being highly sensitive to ‘*Cleaning products such as disinfectants, bleach, bathroom cleansers or floor cleaners’*, and 60% to ‘*Certain perfumes, air fresheners or other fragrances’*(items 6 and 7 on the QEESI’s Chemical Exposures Scale).

### The precautionary principle

Within the last 60 years, thousands of new chemicals have been introduced into our lives and homes (Bernhardt *et al.*, 2017). Little is known about the long-term health effects of low-level chemical exposures on vulnerable people such as infants, the elderly, or those with CI. Increasingly, researchers today suspect exposures to various medications, pesticides, traffic exhaust, plasticizers, and flame retardants, to name a few, could play a role in the increase in CI, developmental disorders, or other chronic conditions.

In the absence of definitive scientific information, expectant mothers, the elderly, those with chronic health conditions, or those with CI can take precautions by reducing unnecessary exposures. The odors of solvents, pesticides, or cleaning products can serve as early warning signs that a potentially harmful chemical is nearby. Table 6 lists potential actions that vulnerable individuals can take to reduce unnecessary exposures to chemicals. Suggested educational resources to help identify and reduce home exposures can be found in the supplemental files S1–S3.

**Table 6.** Precautionary principal

The personal precautionary principle	
Instead of using:	Try using:
Pesticides indoors or on lawns, or mothballs	Baits or traps to control bugs indoors (Avoid attracting bugs by tightly sealing foods, including pet foods)
Paints, varnishes, glues, and polishes with high solvent content	Low-solvent-content paints, water-based finishes and glues (Have these applied when you are away from home)
Bleach, ammonia, disinfectants, and strong cleaning products	‘Elbow grease’, soap and water, baking soda and vinegar
Scented products, perfumes, air fresheners and incense	Unscented cleansers, laundry detergent, fabric softeners,, and cosmetics
Hair coloring, permanents, hair spray, or any aerosol product	New haircut and hair gel or styling products that are unscented and do not require spraying
Dry cleaning, odorous soft plastic toys, or mattress covers	Washable toys, bedding, and clothes
Odorous flooring, such as vinyl, pressed wood or particle board, or carpeting which can also trap allergens	Ceramic, stone tile, or hardwood floors
Commercial foods that may contain pesticides or other questionable ingredients	Organic foods and foods without additives or artificial colors

### Modifiable EHC protocol

Our EHC process is based on observation, measurement, and feedback/education to identify potential exposure triggers in the home – with the goal to reduce symptoms. However, our entire house call protocol as implemented in this study may not be feasible for all situations. For example, a person’s readiness to relinquish usual personal care or cleaning products or the family’s willingness to comply with recommended actions may be barriers. Nevertheless, components of the EHC apply to primary and specialty practices including family and internal medicine, pediatrics, obstetrics-gynecology, geriatrics, and psychiatry. Those physicians can apply the precautionary principle in treating complex and difficult-to-diagnose disorders including asthma, CI, COPD, recurrent pneumonia, chronic fatigue, fibromyalgia, and various neurological conditions. A vigorous 5-visit EHC study is too involved for most practices, but we suggest three alternative levels of EHC interventions that can be customized for different situations (see supplementary material S3).

### Study limitations and strengths

Although we provide good evidence that the greatest symptom changes co-occurred with significant reductions in VOCs, these results should be considered preliminary until replication with a larger sample and a more definitive experimental design is performed. For example, those receiving the EHC were compared to controls who did not receive any contact with the investigators or receive a placebo intervention. Therefore, we were not able to definitively rule out a Hawthorne effect (eg subjects in an experimental study who tend to respond the way they think the investigator wishes).

Further, this study had a small sample size with low power, and as such, did not conform to a standard statistical significance level of .05, and did not correct for multiple comparison tests with statistical methods such as Bonferroni correction. We justify these uncertainties in keeping with the spirit of an investigatory pilot study, where we err on the side of not missing a potentially true effect (eg a Type II error). Therefore, these results should be considered interestingly suggestive but not definitive. Investigations with larger sample sizes are warranted.

Our study’s VOC sampling was limited to ‘grab’ samples at two points in time that provided ‘snapshots’ of indoor air conditions, which may be affected by seasonality (temperature and humidity). Future investigators might use continuous or repeated testing that would better document indoor air exposures over time and pre- and post-intervention.

During pandemics of this nature, IAQ measures are likely compounded during a lockdown due to increased use of disinfectants and/or restricted outdoor airflow. Consequently, the risk of illness is elevated (Domínguez-Amarillo *et al*., 2020). It is important to note that this study was conducted prior to the COVID19 crisis; therefore, the potential bias of additional poor IAQ did not affect our results.

A particular strength of this pilot study is that we were able to associate chemically intolerant participants’ symptom improvement with reductions in measured VOCs in the home. Another strength is the study’s relevance to primary care. We recruited participants from a family medicine practice and used a validated instrument (the QEESI) to characterize our cohort of chemically intolerant participants. By engaging an independent lab for VOC analysis, we were able to identify specific VOCs, including very low-level fragranced terpenes (linalool, limonene, α-pinene, β-pinene) in patients’ homes. This aided our team’s ability to personalize recommendations. It also lessened our concerns about a Hawthorne effect.

## Conclusion

We tend to view our homes as safe havens or refuges. People are often unaware of their exposures, or may adapt to them, and rarely mention any concerns during their medical visits. Indoor air problems simply are not part of most doctors’ differential diagnoses. But our study and prior studies show a high prevalence of CI among people visiting primary care clinics.

Our three-question screening questionnaire – the BREESI – can help physicians identify which patients should complete the QEESI. After using the QEESI to identify patients with CI, the practitioner can help by counseling them regarding their home exposures to VOCs. The future of clinical medicine could include EHCs as standard of practice for susceptible patients. However, primary care clinicians should not wait before addressing the home environment for patients with CI. We are currently exploring the feasibility of conducting virtual EHCs.

## Supplementary material

For supplementary material accompanying this paper visit http://doi.org/10.1017/S1463423621000864. click here to view supplementary material
