# Supplementary material for: Does improving indoor air quality lessen symptoms associated with chemical intolerance?
Source: Prim Health Care Res Dev. 2022 Jan 12;23:e3. doi: 10.1017/S1463423621000864 (PMC8822326; doi:10.1017/S1463423621000864)
Supplement: Supplementary file 1 [file S1463423621000864sup001.docx]

**Supplemental Material**

**S1: Clean Air Oasis**

**
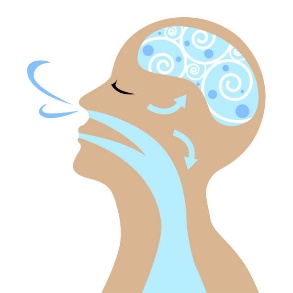
7 Steps to Creating a Clean Air Oasis**

***WWe spend 90% of our day indoors where the air often is more polluted than the air outside.***

ReResearch suggests that a “clean room” may help people who suffer from breathing difficulties, allergies, headaches, brain fog/confusion, fatigue and other health problems. You can create a clean air oasis in your home or in one room, where the air is as free as possible of chemicals, smoke, fragrances, and allergy triggers.

1. **Pick a room**

Choose the room where you spend most of your time, usually your bedroom. Bring in fresh air whenever possible.

1. **Eliminate air pollutants**

Remove all products that have strong odors such as cleaning and laundry products, pesticides, perfume/cologne, scented lotions, deodorants, cosmetics, candles, air fresheners, and aerosols like hair spray which form tiny droplets that are easily inhaled.

1. **Do not permit pets inside the Oasis**

Furry pets can trigger asthma, allergies, and other problems.

1. **Clean safely**

Use only fragrance-free products for cleaning and doing laundry. Cleaning and vacuuming are best done when sensitive individuals are not in the immediate area. Ventilate during and after cleaning.

1. **Avoid burning anything indoors**

Smoke and combustion gases irritate the lungs. Do not permit smoking, vaping, or candle- or incense-burning. Do not use fireplaces, open-flame gas heaters or unvented water heaters. Prevent carbon monoxide poisoning—never heat your home using a gas stove, gas oven, or Hibachi. If you move or purchase new appliances, electric stoves and other appliances are the better health option.

1. **Go the extra mile**

An air purifier with HEPA and charcoal filters can remove pollutants. Keep it running while the room is occupied, including overnight. Bring in fresh outside air whenever possible.

1. **Learn more**

Many of our choices affect the air we breathe indoors. Learn how to remove fragrances from fabrics, stop pests without using pesticides, control humidity and mold, and find safer products for home repair/remodeling. Visit https://makelivesbetter.uthscsa.edu/tilt for more information.

**S2: Suggested educational resources to help identify and reduce home exposures.**

- Environmental Working Group: <https://www.ewg.org/healthyhomeguide/>
  - TILT website: <https://tiltresearch.org/about-tilt/triggers-prevention/>
  - EPA: <https://www.epa.gov/indoor-air-quality-iaq/protect-indoor-air-quality-your-home>
- https://www.epa.gov/indoor-air-quality-iaq/interactive-tour-indoor-air-quality-demo-house
- https://www.epa.gov/indoor-air-quality-iaq/volatile-organic-compounds-impact-indoor-air-quality
- IAQA: https://iaqa.org/consumer-resources/5-easy-tips-to-get-a-healthy-home/
- USDA’s Healthy Homes Partnership: https://extensionhealthyhomes.org/ccontent.html

**S3: We** **suggest three levels of intervention that can be customized for different situations.**

- Level 1: Can be used in any clinic. Administer the 3-item BREESI CI screener (Plos One, 2020) and the QEESI to individuals who might benefit from improved indoor air quality. Provide educational resources to help identify and reduce home exposures and symptom triggers. Periodically, follow up using QEESI Symptom Scale to evaluate symptom improvement.
- Level 2: Plans include the creation of a home environment oasis, an area or room in the home where the patient spends most of the day, typically the bedroom, where air quality can be optimized. Optimization may require HEPA filtration to remove fine particles and activated charcoal filters to remove VOCs.
- Level 3: Used in this study, includes Level 1 protocol, and requires an EHC team trained to take medical and exposure history, conduct home assessments to identify potential exposures and symptom triggers (e.g., fragrance-emitting devices, mold), use portable environmental testing equipment and collect environmental samples for lab analysis. Provide individualized action plans to reduce exposures. Conduct follow-up visits to evaluate action plan compliance and re-assess indoor air and symptoms.
